# Indirect Comparisons: A Review of Reporting and Methodological Quality

**DOI:** 10.1371/journal.pone.0011054

**Published:** 2010-11-10

**Authors:** Sarah Donegan, Paula Williamson, Carrol Gamble, Catrin Tudur-Smith

**Affiliations:** Centre for Medical Statistics and Health Evaluation, University of Liverpool, Liverpool, United Kingdom; Canadian Agency for Drugs and Technologies in Health, Canada

## Abstract

**Background:**

The indirect comparison of two interventions can be valuable in many situations. However, the quality of an indirect comparison will depend on several factors including the chosen methodology and validity of underlying assumptions. Published indirect comparisons are increasingly more common in the medical literature, but as yet, there are no published recommendations of how they should be reported. Our aim is to systematically review the quality of published indirect comparisons to add to existing empirical data suggesting that improvements can be made when reporting and applying indirect comparisons.

**Methodology/Findings:**

Reviews applying statistical methods to indirectly compare the clinical effectiveness of two interventions using randomised controlled trials were eligible. We searched (1966–2008) Database of Abstracts and Reviews of Effects, The Cochrane library, and Medline. Full review publications were assessed for eligibility. Specific criteria to assess quality were developed and applied. Forty-three reviews were included. Adequate methodology was used to calculate the indirect comparison in 41 reviews. Nineteen reviews assessed the similarity assumption using sensitivity analysis, subgroup analysis, or meta-regression. Eleven reviews compared trial-level characteristics. Twenty-four reviews assessed statistical homogeneity. Twelve reviews investigated causes of heterogeneity. Seventeen reviews included direct and indirect evidence for the same comparison; six reviews assessed consistency. One review combined both evidence types. Twenty-five reviews urged caution in interpretation of results, and 24 reviews indicated when results were from indirect evidence by stating this term with the result.

**Conclusions:**

This review shows that the underlying assumptions are not routinely explored or reported when undertaking indirect comparisons. We recommend, therefore, that the quality of indirect comparisons should be improved, in particular, by assessing assumptions and reporting the assessment methods applied. We propose that the quality criteria applied in this article may provide a basis to help review authors carry out indirect comparisons and to aid appropriate interpretation.

## Introduction

Systematic reviews of randomised controlled trials are the highest quality evidence to support healthcare decisions. When the relative effectiveness of interventions is of interest, evidence from trials that compare the interventions directly (head-to-head trials) and evidence from indirect comparisons may be sought within a review. A systematic review of randomised controlled trials that directly (head-to-head) compare two interventions would generally be regarded as the highest quality evidence to support healthcare decisions on the comparative effectiveness of two interventions. In many clinical areas this high quality evidence may not exist or may be inconclusive and utilising alternative sources of evidence such as an indirect comparison could be appropriate. For example, pharmaceutical companies may be reluctant to compare a new drug against the effective standard drug in a head-to-head trial in case results do not favour the new drug. Furthermore, indirect evidence can be more reliable than direct evidence in some cases, for instance, when direct evidence is biased due to the methodological inadequacies of trials that compare the treatments directly [1]. To illustrate an indirect comparison, suppose that the comparison between two interventions, *A* and *B*, is of interest. If both interventions (*A* and *B*) have at some point been compared with a third common intervention (denoted *C*) in separate randomised controlled trials, then an indirect comparison is possible. If trials exist that compare *A* and *B* directly, then direct evidence also exists in addition to the indirect evidence.

Numerous approaches exist to undertake an indirect comparison, a review of which has been undertaken by Glenny *et al*, who recommend that the indirect comparison methodology should preserve the within-trial randomisation [2]. Examples of approaches within this framework include:

- the ‘adjusted’ method by Bucher *et al* [3];
- meta-regression [2];
- hypothesis tests, that test for a difference between treatments effects of *A* relative to *C* and *B* relative to *C* [4], [5];
- examination of the overlap of confidence intervals for treatments effects of *A* relative to *C* and *B* relative to *C* [4].

In contrast, the ‘naive’ method would compare treatment *A* against treatment *B* ignoring treatment *C* and therefore break within trial randomisation. Naive indirect comparison methods are therefore not recommended and are considered to be equivalent to observational data and subject to similar biases [2], [3].

The core assumption underlying indirect comparison methodology is similarity of treatment effects [6]. Thus, the true treatment effect comparing any two interventions would be similar across all trials irrespective of whether they included one or both of those interventions. If the similarity assumption is violated, the validity of the result of the indirect comparison is questionable. Since the treatment effect *A* relative to *C* is not actually observed in the *B* vs. *C* trials (except when three-arm trials are included), the similarity assumption is difficult to assess. No well-established methods exist to determine when the similarity assumption holds; however, comparing patient or trial characteristics across the trials involved in the indirect comparison, and investigating the effect of patient or trial characteristics on the indirect comparison result using subgroup analysis, sensitivity analysis, or meta-regression, may indicate whether similarity is reasonable [7].

Other key assumptions that underlie indirect comparison methodology are homogeneity and consistency. Homogeneity concerns the similarity within the head-to-head *A* vs. *C* trials, and the similarity within the head-to-head *B* vs. *C* trials. Standard methods to assess homogeneity exist [4]. Consistency refers to the similarity of direct and indirect evidence for the same treatment comparison. Methods to assess consistency for indirect comparisons have been proposed [2], [8], [9].

The assumptions of similarity, homogeneity and consistency can be thought of as an extension of the usual homogeneity assumption in standard meta-analysis. Assessment of the assumptions is vital to ensure the results of indirect comparisons are valid and interpreted appropriately. Since no guidelines concerning the reporting of indirect comparisons and assessment methods exist, the importance of a review of the reporting and methodological quality of the indirect comparison methods applied in published reviews is clear.

Existing research articles have summarised the indirect comparison methodology applied in published reviews and relevant methodological problems. Recently, Song et al published a summary of methodological problems identified by surveying published reviews of mixed treatment comparison meta-analysis. The methodological problems reviewed were: the mixed treatment comparison method used; whether the similarity assumption and consistency assumption was mentioned; whether efforts were made to investigate or improve the similarity for mixed treatment comparisons; and whether direct and indirect evidence was combined or compared [6]. Additionally, Edwards et al searched for systematic reviews that included indirect comparisons of treatments and methodological articles concerning indirect comparisons. The various indirect comparison methods applied in the published reviews were summarised along with discussion about the pros and cons of each specific method [10]. Also, Glenny *et al* searched for reviews that applied indirect comparison methodology and summarised the methods and results of the reviews [2].

The primary aim of this article is to report a systematic review of the reporting and methodological quality of published indirect comparisons using specifically devised quality assessment criteria. These criteria may provide a basis for the future development of a quality assessment tool for the evaluation and critical appraisal of indirect comparisons to aid appropriate interpretation. The review also adds empirical data to the existing evidence and highlights further the importance of improving reporting quality with some preliminary recommendations made.

## Methods

### Eligibility criteria

Inclusion Criteria:

- Reviews that applied statistical methods to indirectly compare the clinical effectiveness of two interventions (*A* and *B*) based on randomised controlled trials.
- An intervention is defined to be any treatment, dose, treatment regimen, or clinical procedure.
- A review was considered to have applied statistical methods to make an indirect comparison when a quantitative summary of the indirect comparison of two interventions was produced or a description of the overlap of confidence intervals was given.
- An individual review may include more than one indirect comparison of two interventions provided separate analyses were undertaken and presented.

Exclusion Criteria:

- Review protocols or abstracts.
- Methodological publications that presented indirect comparisons for illustrative purposes.
- Cost effectiveness reviews.
- Narrative reviews of trials, meta-analyses, treatment policies, or available treatments.
- Reports of a single trial.
- Reviews that did not compare interventions (e.g. reviews that compared different populations of patients).
- Indirect comparisons based on non-randomised trials.
- Reviews that indirectly compared interventions qualitatively (i.e. did not apply statistical methods).
- Reviews that indirectly compare more than two interventions simultaneously (for example using mixed treatment comparison meta-analysis).

### Search strategy

The following databases were searched using specific search terms (Table S1): The Database of Abstracts and Reviews of Effects (DARE) (1994 to March 2008), The Cochrane library (March 2008), and Medline (1966 to March 2008). Reviews were sought regardless of language. Duplicate citations were excluded.

### Review selection

The full publication was obtained for each review located by the search and independently assessed against the eligibility criteria by two reviewers using an eligibility form. After assessment, differences in the assessment results were discussed. Reports were scrutinised to ensure that only the latest version of updated reviews was included.

### Data extraction

Information was extracted using a data extraction form regarding: general characteristics of the reviews, such as, the inclusion criteria in terms of patients, interventions, trial design, and primary outcomes; the indirect and direct comparisons made; the number of trials and patients in the indirect comparison; the type of data and measure of effect for the primary outcome; and whether the review was based on individual patient data or aggregate data.

We also extracted information regarding the indirect comparison method; the consideration and assessment of the similarity, homogeneity, and consistency assumptions; reporting of results; and interpretation of the evidence. More specifically, we extracted the indirect comparison method reported or applied and the type of results presented (e.g. measure of effect, confidence interval, p-value, number of trials, number of patients). Regarding the similarity assumption, we extracted information such as: the assumption's phrasing; any reported assessment methods; whether sensitivity analysis, subgroup analysis, or meta-regression was applied to investigate if the indirect comparison result varied; any remarks regarding the results of such methods; and whether patient or trial characteristics across all trials included in the indirect comparison were reported, compared, or comparable. For the homogeneity assumption, we extracted details such as: the assumption's phrasing; the assessment method reported or applied; whether the homogeneity assumption was satisfied based on quantitative results or concluding statements; whether a fixed effects or random effects model was applied; whether sensitivity analysis, subgroup analysis, or meta-regression was applied across trials in each trial set involved in the indirect comparison; and any remarks regarding the results of these methods. Regarding the consistency assumption, we extracted information such as: the assumption's phrasing; the assessment method reported or applied; whether the assessment method was satisfied based on quantitative results or concluding statements; whether direct and indirect evidence was combined and the type of results presented (e.g. measure of effect, confidence interval, p-value, number of trials, number of patients); whether patient or trial characteristics across all trials were reported, compared, or comparable; whether three-arm trials were included using direct evidence rather than indirect evidence from the trial; reasons given for using indirect and direct evidence; and whether results from each head-to-head trial were reported. For reporting of results we extracted details, such as, whether the meta-analytic result for each of the two trial sets involved in the indirect comparison was presented and the type of results given (e.g. treatment effect estimate, confidence interval, p-value, number of trials, number of patients); whether results from all the individual trials' were reported and the type of results given (trial arm summary data or treatment effect estimates); and whether the review indicated which results were based on indirect evidence. Regarding interpretation we extracted information, such as, whether the review indicated that direct and indirect evidence are not equivalent; and whether the review indicated that more head-to-head trials were needed.

The data extracted related to the review's primary outcome where stated, or the outcome for which results were reported first in the absence of a specific primary outcome. When reviews did not specifically report the number of trials (or patients) in the indirect comparisons the number of trials (or patients) were calculated based on the data from direct comparisons. The review author was not contacted in the case of unclear or missing data as it was considered that the quality assessment should be based solely on the reported information.

### Data analysis and quality assessment

The general characteristics of reviews were summarised. Categorical data were summarised using frequencies.

We independently assessed the quality of the reporting and application of indirect comparison methods in each review using specific quality criteria. Differences in the assessment results were discussed. Initially, the criteria were compiled from recommendations given in published literature [1]–[5], [8], [9], [11]. The feasibility of the assessment was tested by one author by pre-piloting the initial criteria. The criteria were then condensed and adapted to focus on the main points of interest. For example, we disregarded a criterion that considered whether the indirect comparison method had been specifically reported in the review and instead focussed on whether the method applied maintained randomisation by recalculating the indirect comparison to determine the method applied or otherwise (see criterion 1 in Table 1). The final criteria focus on six quality components: indirect comparison method; consideration and assessment of the similarity, homogeneity, and consistency assumptions; reporting of results; and interpretation of evidence. The final criteria are displayed in Table 1. Reviews were classified as yes (representing higher quality), no (representing lower quality), or unclear for each criterion. The proportions and percentage of reviews were calculated for each classification for each criterion.

**Table 1:** Summary of quality assessment criteria.

Criteria	Yes (%)	No (%)	Unclear (%)	Not applicable
**Indirect comparison method**				
Is the method applied to undertake the indirect comparison adequate?(1)	41 (95)	2 (5)	0 (0)	0
If an adequate method is used, is a treatment effect estimate and measure of precision reported?	25 (61)	16 (39)	0 (0)	2
**Similarity**				
Is the assumption of similarity stated?	11 (26)	32 (74)	0 (0)	0
Is a method described to assess the similarity assumption within the review methods section?(2)	0	43 (100)	0 (0)	0
Is a reasonable approach used to assess the assumption of similarity?(3)	19 (44)	22 (51)	2 (5)	0
Are patient or trial characteristics reported for all trials in the indirect comparison?	38 (88)	5 (12)	0 (0)	0
Are patient or trial characteristics compared across the two trial sets involved in the indirect comparison?	11 (26)	32 (74)	0 (0)	0
Are patient or trial characteristics reported to be comparable for the two trial sets involved in the indirect comparison?	4 (9)	5 (12)	34 (79) (2 unclear if comparable; 32 not reported)	0
**Homogeneity across trials within each of the two trial sets involved in the indirect comparison**				
Is the method used to determine the presence of statistical heterogeneity adequate?(4)	24 (60)	12 (30)	4 (10)	3
Is the homogeneity assumption satisfied or is statistical heterogeneity accounted for if present?(5)	19 (48) (8 homogeneous; 11 accounted)	3 (8)	18 (45) (17 unclear if homogeneous; 1 unclear if accounted)	3
If the homogeneity assumption is not satisfied, is clinical or methodological homogeneity across trials in each trial set involved in the indirect comparison investigated by an adequate method?(6)	12 (38)	19 (59)	1 (3)	11
**Consistency**				
Is consistency of effects assessed?^(7)^	6 (35) (1used statistical method)	11 (65)	0 (0)	26
If the direct and indirect evidence is reported to be consistent, is the evidence combined and the result presented?(8)	1 (25)	3 (75)	0 (0)	39
If inconsistency is reported, is this accounted for by not combining the direct and indirect evidence?(9)	2 (100)	0 (0)	0 (0)	41
Are patient or trial characteristics compared between direct and indirect evidence trials?^(10)^	5 (29)	12 (71)	0 (0)	26
Are patient or trial characteristics for direct and indirect evidence trials reported to be comparable?^(11)^	2 (12)	1 (6)	14 (82) (2 unclear if comparable; 12 not reported)	26
Are any included 3-arm trials correctly analysed?(12)	3 (25)	9 (75)	0 (0)	31
Is justification given for using indirect evidence and direct evidence?^(13)^	8 (47)	9 (53)	0 (0)	26
Does the review present results from all trials providing direct evidence ?^(14)^	(65)	6 (35)	0 (0)	26
**Interpretation**				
Is a distinction made between direct comparisons and indirect comparisons?	25 (58)	18 (42)	0 (0)	0
Does the review state that more trials providing direct evidence are needed?	24 (56)	19 (44)	0 (0)	0
**Reporting**				
Does the review present both of the meta-analysis results from each of the two trial sets involved in the indirect comparison?	37 (86)	6 (14)	0 (0)	0
Was it highlighted which results were from indirect evidence?(15)	24 (56)	19 (44)	0 (0)	0
Are the individual trials' treatment effect estimates reported?	23 (53)	20 (47)	0 (0)	0

(1) Yes: method preserves randomization. No: method does not preserve randomization.

(2) Yes: reported a method that is stated will assess the assumption of similarity. No: do not report a method.

(3) Yes: sensitivity analysis, subgroup analysis, or meta-regression used to assess consistency of the indirect comparison across different trial or patient characteristics. No: no method, no analysis that includes all the trials in the indirect comparison. Unclear: unclear if the trials used in the analysis are the same trial sets involved in the indirect comparison.

(4) Yes: Chi-square test, I-squared statistic, estimating the between trial variance from a random effects models. No: no method applied, or not applied to the two trial sets contributing to the indirect comparison. Unclear: unclear if heterogeneity was assessed for the two trial sets contributing to the indirect comparison. Not applicable: only one trial in each trial set.

(5) Yes: no heterogeneity present (reported by authors or determined from the results), or accounted for heterogeneity using the random effects model. No: heterogeneity not accounted for using the random effects model. Unclear: unclear if heterogeneity present, or unclear if heterogeneity taken into account using the random effects model. Not applicable: only one trial in each trial set.

(6) Yes: sensitivity analysis, subgroup analysis, or meta-regression used to assess homogeneity across different trial or patient characteristics within each of the two trials sets involved in the indirect comparison. No: no method, no analysis that includes the trials in each of the two trial sets involved in the indirect comparison. Unclear: unclear if the trials used in the sensitivity analysis, subgroup analysis, or meta-regression are the same set of trials as those in each of the two trial sets involved in the indirect comparison. Not applicable: only one trial in each trial set; or homogeneity assumption satisfied.

(7)–(14) Not applicable: both indirect and direct evidence are not presented for the same comparison.

(8) Not applicable: reported to be inconsistent, or unclear if consistent based on text or results.

(9) Not applicable: reported to be consistent, or unclear if inconsistent based on text or results.

(12) Yes: three-arm trials are correctly analysed i.e. indirect evidence (*AC, BC*) is not included and direct evidence (*AB*) is analysed, and data from a three-arm trial is not combined as though it is from two different studies. No: three-arm trials are incorrectly analysed i.e. indirect evidence (*AC, BC*) is included and direct evidence (*AB*) is not analysed, or data from a three-arm trial is combined as though it is from two different studies. Na: no three-arm trials are included in the review.

(15) Yes: the term indirect comparison is stated when referring to the result or the result is presented under a heading that states the result is based on an indirect comparison. No: result is presented without noting it is an indirect comparison.

We considered higher quality reviews to be reviews that applied indirect comparison methods that preserved randomisation and that presented a measure of treatment effect and measure of precision. Regarding similarity, reviews were classed as higher quality when they stated the similarity assumption and a method to assess the assumption; applied a suitable assessment method, such as, sensitivity analysis, subgroup analysis or meta-regression including all the trials in the indirect comparison; and presented and compared patient or trial characteristics for all trials in the indirect comparisons. Regarding homogeneity, we considered higher quality reviews to be reviews that applied a suitable method to assess homogeneity (such as the chi-square test, I-square statistic, or estimating the between trial variance in a random effect model) and if heterogeneity was evident that it was accounted for using a random effects model and explored using sensitivity analysis, subgroup analysis or meta-regression. Regarding consistency, reviews were classed as higher quality when they assessed consistency; did not combine indirect and direct evidence in the presence of inconsistency; and compared patient or trial characteristics for all trials contributing direct and indirect evidence. We classed reviews that included three arm trials as lower quality when the review author ignored the direct evidence in the trial. We classed reviews as higher quality when justification for including direct and indirect evidence was given; and when the results from trials contributing direct evidence were presented. Regarding interpretation, we considered reviews to be of higher quality when the review author explained that direct and indirect comparisons are not equivalent to avoid misinterpretation of the results; and when the review author considered the strength of direct evidence. For reporting, reviews were judged to be of higher quality when the review author presented the two meta-analytic results used in the indirect comparison and the individual trials' results; and when the review author indicated when results of indirect comparisons were reported.

## Results

**Figure 1:**
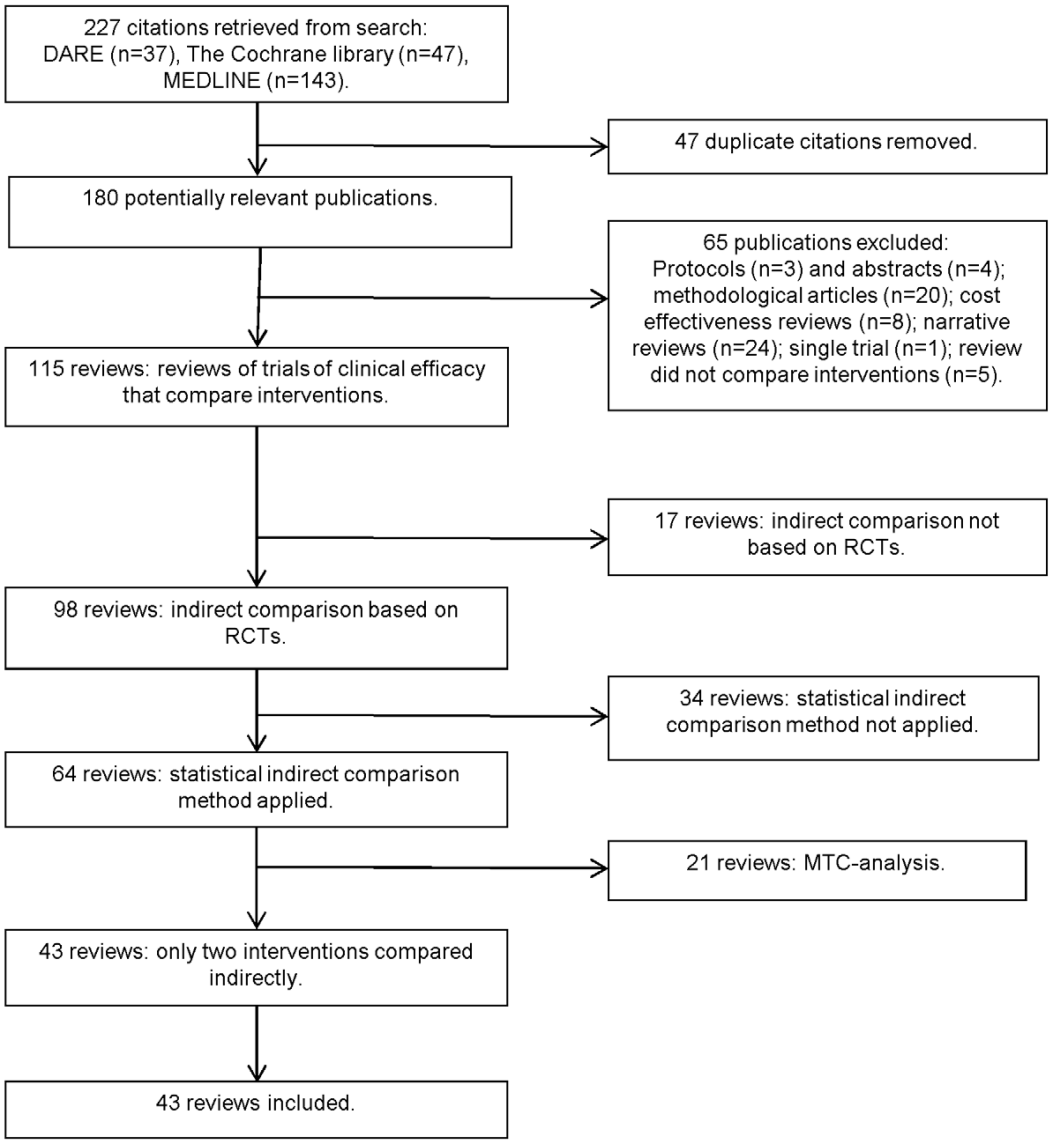
Selection process for reviews. Abbreviations: RCTs (randomised controlled trials).

**Figure 2:**
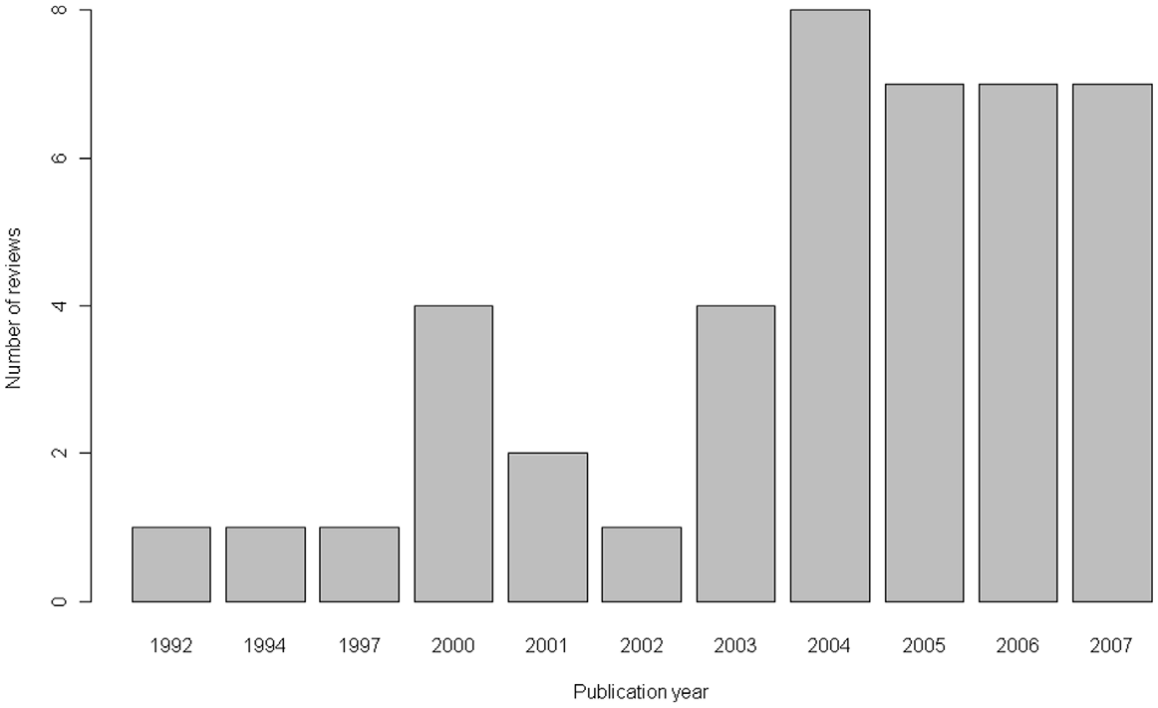
Frequency of published reviews including indirect comparisons, by year of publication.

Figure 1 displays the review selection process. The 43 included reviews were published in 35 English language journals between 1992 and 2007 (Figure 2) [12]–[54].

See Table S2 for the characteristics of included reviews.

Most indirect comparisons (30 reviews) were based on fewer than 15 trials. The indirect comparisons made are reported in Table S2.

Reviews were focussed in a variety of clinical areas: circulatory (11 reviews); musculo-skeletal (nine reviews); reproductive (four reviews); HIV (three reviews); psychological (three reviews); cancer (two reviews); gastrointestinal (two reviews); post-operative (two reviews); psychiatric (two reviews); diabetes (one review); ocular (one review) and other clinical areas (three reviews). A range of outcomes were studied in the reviews as described in Table S2.

Dichotomous outcome data predominated (32 reviews) with treatment effects summarised using the risk ratio (16 reviews), the odds ratio (13 reviews), or the risk difference (three reviews); continuous outcomes were presented using the mean difference (six reviews) and the standardised mean difference (three reviews); count data were reported using the rate ratio (one review); and time to event data were summarised with the hazard ratio (one review). One review stated that individual patient data were analysed but the remainder were based on aggregate data.

A variety of interventions were compared indirectly. Thirty-four reviews indirectly compared pharmacological treatments: drugs (20 reviews), doses or regimens (seven reviews), and drug combinations (seven reviews). Nine reviews compared non-pharmacological interventions: vitamin/mineral supplements (two reviews), testing methods (two reviews), and treatment delivery (five reviews).

### Quality assessment

Table 1 displays the quality assessment results. Refer to Table S3 for the quality assessment results for each criterion for each review.

#### Indirect comparison methodology

Adequate statistical methods, that is, methods that preserved randomisation within trials, were applied in 41 reviews (95%): 23 reviews applied the adjusted method, six reviews used meta-regression, five reviews compared the overlap of confidence intervals, and seven reviews used significance tests. Two reviews (5%) applied inadequate methods (naïve method).

Of the 41 reviews that used adequate methods, only 25 (61%) presented a measure of treatment effect and its precision for the indirect comparison (22 used the adjusted method, three used meta-regression).

#### Similarity

The similarity assumption was stated in 11 reviews (26%) using various terminology and described in different sections of the review manuscript; the assumption was described in the introduction (one review), methods (two reviews), results (two reviews), discussion (five reviews), and appendix (one review) (Table 2).

**Table 2:** Phrasing of the similarity assumption.

Review	Phrasing of the similarity assumption
Berner 2006	“The chosen procedure bases on the assumption, that agents are comparable through their relative effect vs. a common comparator” (methods).
Boonen 2007	“The validity of an adjusted indirect comparison depends on the internal validity of the RCTs involved. The methodology assumes similarity in trial design and methodological quality. Another assumption is that the magnitude of the treatment effect is consistent in patients across different trials” (discussion).
Chou 2006	“that the relative effect of one treatment compared with another is consistent across the entire set of trials” (introduction).
Clark 2004	“For the adjusted indirect comparison to be valid, the key underlying assumption is that the relative efficacy of an intervention is consistent in patients included in different trials; that is, that the estimated relative efficacy is generalisable” (results).
Collins 2007	“However, this method is only valid when the magnitude of the treatment effect is consistent between the different studies being compared” (appendix).
Hochberg 2003	“The authors did note that there were several assumptions that should be fulfilled in order to support the inferences drawn from these comparisons, including similarity of methodology in trial design and measurement of clinically important outcomes, and consistency of treatment effect in different subgroups of patients” (discussion).
Jones 2004	“However, the method is only valid when the magnitude of the treatment effect is consistent between the different studies being compared” (results).
Lim 2003	“The validity of indirect comparison meta-analysis is built on the assumption that no important differences exist between trials examining medium or low dose regimens. If the two sets of trials differ with respect to a feature (clinical or methodological) that modified the treatment effect, then the comparisons of medium and low dose aspirin would be confounded” (discussion).
Otoul 2005	“In order for this indirect comparison to be valid, the overall characteristics of the trials included in the meta-analyses could not differ systematically. The main statistical assumption in this adjusted method is that the relative effect of a drug is consistent; i.e., the odds ratio is the same in patients included in different trials” (methods).
Sauriol 2001	“The indirect approach to meta-analysis requires certain conditions to yield optimal results. For example, it is important that study designs, patient inclusion/exclusion criteria, and patient characteristics at baseline are as similar as possible across studies. Heterogeneity in study design or study population can lead to heterogeneity in results, and may lead to nonvalid conclusions. Therefore, the use of such methods does not always lead to the same conclusions” (discussion).
Zhou 2006	“The presence of clinical heterogeneity in these trials was evident; however, results from meta-analysis and substudies, particularly those using individual patient data, have indicated that the RR reduction of cardiovascular events by statin did not depend on the patients' risk stratified by age, sex, CHD history, and other cardiovascular risk factors. This consistency in the effect across different baseline characteristics is also required by the method of adjusted indirect comparison to ensure valid results” (discussion).

None of the reviews explicitly described a method to examine the assumption of similarity within the methods section. However, 19 reviews (44%) did apply reasonable methods to explore this assumption:

- grouping the trials according to a particular characteristic, indirectly comparing interventions for each grouping (i.e. subgroup analysis) (seven reviews);
- conducting meta-regression including trial-level summaries that may modify the treatment effect (four reviews);
- selecting a trial group based on a particular characteristic and indirectly comparing interventions using the selected trial subset (i.e. sensitivity analysis) (eight reviews).

Analyses varied greatly in terms of the number of variables studied.

A summary of patient and trial characteristics were presented in 38 reviews (88%), although the number of characteristics varied substantially across reviews. Only eleven reviews (26%) compared characteristics between the two trials sets contributing to the indirect comparison: four reviews reported that characteristics were comparable; five reviews stated characteristics were dissimilar (characteristics described as being dissimilar included: study duration, disease severity, dose, and outcome definition) but continued to estimate the indirect comparison; and two reviews did not state whether or not characteristics were comparable, thus were unclear regarding comparability, but did discuss the similarities and differences of characteristics among the trials.

#### Homogeneity

Three reviews included one trial per treatment comparison therefore homogeneity assessment was not applicable. To determine the presence of heterogeneity, 24 reviews (60%) implemented adequate methods, namely the Chi-squared test, I-squared statistic, or estimation of between-trial variability. Twelve reviews (30%) did not report an adequate method or results of a homogeneity assessment for the relevant group of trials. The assessment method was unclear or it was unclear whether the assessment had included the group of trials of interest in four reviews (10%).

Based on the I-squared statistic, Chi-square test, or statements reported, the homogeneity assumption seemed reasonable in eight reviews. There was evidence of heterogeneity in 15 reviews, 11 of which applied a random effects model. In seventeen reviews homogeneity was not tested or reported, hence the presence of homogeneity was unclear.

For the 32 reviews for which statistical heterogeneity may exist, twelve reviews (38%) implemented adequate methods: subgroup analysis (seven reviews), sensitivity analysis (two reviews), or meta-regression (three reviews) to explore clinical and/or methodological factors as a potential explanation of statistical heterogeneity within the trial sets. Nineteen reviews (59%) did not explore potential causes of heterogeneity for relevant trial groups. One review (3%), classified as unclear, did not indicate the trial set on which the assessment was applied.

#### Consistency

Seventeen reviews (40%) included direct and indirect evidence in the review for the same comparison. Six of these reviews (35%) assessed consistency of the treatment effects: one review used a hypothesis test to compare the direct and indirect estimates of treatment effect; and five reviews discussed the consistency of direct and indirect treatment effects. Eleven of the reviews (65%) did not assess consistency of the treatment effects.

Of the six reviews that did evaluate consistency, four reported consistent evidence and two reported inconsistency. One review that reported consistency combined direct and indirect effect measures using meta-analysis to produce a pooled effect estimate. Both of the reviews that reported inconsistency investigated differences and did not combine evidence types.

Patient and trial characteristics were compared across direct and indirect evidence trials in five reviews (29%) of which two reported comparability, one reported non-comparability, and two did not report results.

Twelve reviews included information from three-arm trials, but only three of these reviews (25%) correctly analysed these data as direct evidence rather than indirect evidence.

Justification for including indirect evidence and direct evidence was provided in eight reviews (47%), reasons were: limited number of trials providing direct evidence (five reviews), aimed to compare direct and indirect evidence (two reviews), and both reasons (one review).

Six reviews (35%) did not present the results from each trial contributing direct evidence.

#### Interpretation

Twenty-five reviews (58%) made a distinction between indirect comparisons and direct comparisons. Twenty-four reviews (56%) stated that more direct evidence trials were needed.

#### Reporting

Thirty-seven reviews (86%) presented meta-analysis results from each of the two trial sets involved in the indirect comparison. Twenty-four reviews (56%) highlighted when the result was an indirect comparison by stating this term with the result. The treatment effect estimated from each trial was reported in 23 reviews (53%).

## Discussion

### Recommendations to review authors

Guidelines for reporting conventional pair-wise meta-analyses and for producing high quality systematic reviews are already available [4], [55]. This review identifies a clear need to extend such guidelines to indirect comparisons, focussing on the assessment of the underlying assumptions. The quality criteria applied in this article may provide a basis for the future development of a quality assessment tool for the evaluation and critical appraisal of indirect comparisons to aid appropriate interpretation. Key recommendations based on published literature [1]–[5], [8], [9] and expert opinions are given below to help review authors carry out indirect comparisons and to aid appropriate interpretation.

Firstly, the method of analysis, the assumptions made and methods for assessing the plausibility of assumptions, particularly that of similarity should be clearly stated within the methods section of the report. We found that 11 reviews (26%) stated the similarity assumption and even fewer reviews stated the homogeneity assumption and consistency assumption. No review explicitly mentioned the use of a particular method to assess the assumption of similarity.

Although a formal statistical test for similarity is not available, there are approaches that can be used to assess how reasonable is the assumption. The similarity assumption holds when the true treatment effects comparing any two interventions (i.e. *A* vs. *C, B* vs. *C,* and *A* vs. *B*) would be similar across all trials irrespective of which interventions where included in the trial (*A*, *B,* or *C*). If the true treatment effect comparing any two interventions is modified by a particular trial or patient characteristic and all the trials involved in the indirect comparison are not alike with respect to the characteristic, then the assumption will be violated. One approach to assess the similarity assumption is to compare patient characteristics and trial features descriptively across all trials contributing to the indirect comparison. This can help identify variability in any important characteristic that could modify the treatment effects and hence violate the similarity assumption. If characteristics are similar, the similarity assumption is more likely to hold than if characteristics are dissimilar. However, if characteristics vary but are not expected to modify treatment effects then the assumption may still be satisfied. This of course assumes that there are no unknown characteristics that would modify the result. The characteristics studied should be chosen using expert, evidence-based information, as should be the case in any standard meta-analysis. In our review, only 11 reviews (26%) undertook some kind of comparison of trial or patient characteristics. Bucher *et al* compared characteristics across the two trial sets (*A vs. C* and *B vs. C*) by calculating a summary measure for each of the trial sets and then comparing the summary measures [3]. No review followed the method as applied by Bucher *et al.* Secondly, the potential for modification of the result of the indirect comparison can be explored using appropriate characteristics by sensitivity analyses, subgroup analysis, or meta-regression, although the usual limitations of these methods should be kept in mind [56]. Nineteen reviews (44%) applied these methods in an attempt to assess treatment effect modification.

Homogeneity should be assessed within the two trial sets that contribute to the indirect comparison using the same methods as for standard meta-analysis [4]. Statistical heterogeneity is assessed by visually inspecting forest plots, using the Chi-square test, I-squared statistic, and by interpretation of the between trial variance estimate from a random effects model. Overall, only 24 reviews (60%) reported methods to assess statistical heterogeneity or presented the results of such methods. Potential clinical and methodological explanations for statistical heterogeneity can be assessed using subgroup analysis, sensitivity analysis, or meta-regression. In total, 19 reviews (59%) for which heterogeneity was detected, did not investigate heterogeneity using these methods. Patient characteristics and trial features should also be compared across trials within each trial set. We found three reviews for which a fixed effects model was adopted even though statistical heterogeneity was evident. When high levels of unexplained statistical heterogeneity exists a random effects model to account for heterogeneity is more appropriate, or may even indicate that meta-analysis is not appropriate.

Consistency between direct and indirect evidence from two-arm trials can be assessed by comparing characteristics of direct and indirect evidence trials and by using a hypothesis test to indicate whether there is a significant discrepancy between the treatment effect estimates calculated from each evidence type although the test has low power [2], [3], [8], [57]. We found one review (6%) of the 17 that had included direct and indirect evidence that applied this method. A further five reviews (30%) assessed consistency using an unspecified method. It is important that the cause of inconsistency is investigated. Inconsistent evidence may signify bias from methodological inadequacies in the direct or indirect evidence, clinical diversity across patients or a combination of both [1]–. Song *et al* showed that in some cases indirect evidence is less biased than direct evidence [1]. Often the cause of inconsistency means that combining direct and indirect evidence would be inappropriate. We found two reviews that reported inconsistency and neither review combined evidence which is entirely reasonable. When evidence is consistent, the generic inverse variance method can combine direct and indirect evidence; however, the treatment effect estimates from each evidence type should also be reported separately for transparency. We found that four reviews reported consistency and one of these combined the evidence.

With regard to interpretation, since indirect evidence is not the same as direct evidence, this distinction should be stated to avoid misinterpretation. We found 18 reviews (42%) that did not make this distinction. When interpreting indirect evidence, consideration should be given to the generalisability of the patients included in the trials involved in the indirect comparison, just as the generalisability of patients included in trials in a direct comparison should be considered when interpreting direct evidence. Moreover, the results of the assessment of the assumptions can help determine the reliability of the indirect evidence; if the assumptions appear reasonable, the indirect evidence should be valid. In the same way, the assessment of the homogeneity assumption can help determine the reliability of the direct evidence.

The results of the indirect comparison, direct comparison, individual trial results, and the meta-analytic treatment effects from each of the two trial sets involved in the indirect comparison, should be reported. Also, review authors should clearly indicate which results are based on indirect evidence; our findings showed that 19 reviews did not make this indication.

One important aspect not examined in this review is that indirect comparisons should be based on meta-analysis results which are a component of a systematic review as for any other meta-analysis. The usual rigorous methodology and assessment of risk of bias should be undertaken as part of the systematic review [58].

### Comparison with existing evidence

The recently published article by *Song et al* included 88 reviews, substantially more than the 43 reviews included in this overview [6]. However, 14 reviews are included in this article that were not included by *Song et al.* Similarly, 58 reviews were included by Song *et al* which are not included in this assessment. The reason for this disparity is partly due to differences in eligibility criteria, search strategies and search terms. Even so, the results of this review mostly support the findings of Song *et al* but consider the quality of aspects in more depth than previous research. Song *et al* found that trial similarity was discussed or explicitly mentioned in 45% of reviews, where as we found that 26% of reviews explicitly stated the assumption. Song *et al* reported that 26% reviews carried out subgroup or meta-regression to identify or adjust for possible treatment effect modifiers; we found that 44% of reviews undertook similar methods. We found that 26% of reviews compared trial and patient characteristics across all the trials used in the indirect comparison; Song *et al* stated that 30% of reviews compared characteristics. Consistency of direct and indirect evidence was assessed in 71% of reviews that applied the naive approach or adjusted indirect comparison method as described by Song *et al,* where as we established that 35% assessed the consistency of evidence. Song *et al* found that 12% of these reviews combined direct and indirect evidence; we found that 6% of reviews combined evidence.

Song *et al* highlighted the methodological flaws in published indirect comparisons and made recommendations regarding suitable methodology. Our review identifies the importance of improving reporting quality and adds empirical data to the existing evidence regarding methodological quality. The specifically devised quality assessment criteria applied in this review provides a grounding to help review authors carry out indirect comparisons and to aid appropriate interpretation.

### Limitations

The main limitation of this review is that generalisability is restricted because reviews that compared more than two interventions simultaneously, for example, using mixed treatment comparison meta-analysis models were excluded because additional quality criteria and search terms would apply to these reviews. Detailed quality assessment criteria would include modelling details such as allowance for multi-arm trials, specification of variance structures, and assessment of consistency of indirect evidence using different common interventions (that is, different loops of evidence in a treatment network). For this reason, reviews that compared more than two interventions simultaneously will be considered separately following a search using adapted search terms. In total, 21 reviews of randomised trials using mixed treatment comparison methodology were excluded from this overview. However, it is worth noting that the methodology for undertaking a simple indirect comparison is much more accessible than for complex mixed treatment comparisons and therefore widely applicable. Interestingly, Song *et al* reported that 63% of reviews applied the adjusted method or naive approaches, where as only 20% of reviews compared multiple treatments simultaneously using meta-analysis. These results show that this article is applicable to the main body of published reviews.

A further limitation of this review is that we may not have retrieved all published reviews including an indirect comparison because some reviews may not have been indexed using the search terms specified. However there is no reason to believe that the reviews we identified would differ to those we did not identify and hence our sample should be a representative sample of published indirect comparisons in the medical literature. In fact the conclusions reached in this article are comparable to the article by Song *et al* although slightly different sets of reviews were included in each article.

Lastly, a thorough assessment of quality would require clinical knowledge of the individual review topic areas. Clinical knowledge would allow assessment of the similarity assumption as potential patient characteristics that could influence the result of the indirect comparison may be known to those working within the individual review areas.

In conclusion, indirect comparisons can be extremely valuable and their use is increasing in the literature. However, the validity of the indirect comparison relies on underlying assumptions similar to standard meta-analysis. This review shows that these assumptions are not routinely explored when undertaking and reporting indirect comparisons. We recommend therefore, that the methodological and reporting quality of indirect comparisons should be improved and propose that the quality criteria applied in this article may provide a basis to help review authors carry out indirect comparisons and to aid appropriate interpretation.

## Supporting Information

Table S1 Search terms for databases.
(0.06 MB DOC)

Table S2 Characteristics of included reviews. *Outcome name (primary outcome or main outcome); data type; measure of effect. **NT: number of trials; NP: number of patients.
(0.21 MB DOC)

Table S3 Quality assessment results for individual reviews. Abbreviations: c: comparable/consistent, f: fixed effects model, h: homogeneity reported or determined from results, he: heterogeneity reported or determined from results, n: no, na: not applicable, nc: not comparable/consistent, nr: not reported, nt: no three-arm trials, r: random effects model, t: using a statistical test, u: unclear, y: yes.
(0.18 MB DOC)
