# Supplementary material for: Indirect Comparisons: A Review of Reporting and Methodological Quality
Source: PLoS One. 2010 Nov 10;5(11):e11054. doi: 10.1371/journal.pone.0011054 (PMC2978085; doi:10.1371/journal.pone.0011054)
Supplement: Table S1 — Search terms for databases. (0.06 MB DOC) [file pone.0011054.s001.doc]

|  | **DARE** | **Cochrane Library** | **Medline (via OVID)** |
| --- | --- | --- | --- |
| 1 | Randomized controlled trials (MESH) | RANDOMIZED CONTROLLED TRIALS (MESH) | RANDOMIZED CONTROLLED TRIALS/ |
| 2 | controlled clinical trials (MESH) | controlled clinical trials (MESH) | controlled clinical trials.sh. |
| 3 | CLINICAL TRIALS (MESH) | CLINICAL TRIALS (MESH) | CLINICAL TRIALS/ |
| 4 | “Randomi* control* trial*” | Randomi* control* trial* | randomi$ control$ trial$.tw. |
| 5 | “Clinical trial*” | clinical trial* | clinical trial$.tw. |
| 6 | Trial* | Trial* | trial$.tw. |
| 7 | #1 or #2 or #3 or #4 or #5 or #6 | #1 or #2 or #3 or #4 or #5 or #6 | 1 or 2 or 3 or 4 or 5 or 6 |
| 8 | REVIEW, LITERATURE (MESH) | REVIEW, LITERATURE (MESH) | REVIEW LITERATURE/ |
| 9 | Meta-analysis (MESH) | meta-analysis(MESH) | meta-analysis.sh. |
| 10 | Meta-analy* | meta-analy* | meta-analy$.tw. |
| 11 | Metaanaly* | Metaanaly* | metaanaly$.tw. |
| 12 | “Meta analy*” | (meta NEXT analy*) | (meta adj analy$).tw. |
| 13 | #8 or #9 or #10 or #11 or #12 | #8 or #9 or #10 or #11 or #12 | 8 or 9 or 10 or 11 or 12 |
| 14 | “indirect NEAR comparison*” | (indirect NEAR/2 comparison*) (all text) | (indirect adj2 comparison$).tw. |
| 15 | “indirect NEAR evaluat*” | (indirect NEAR/2 evaluat*) (all text) | (indirect adj2 evaluat$).tw. |
| 16 | “indirectly NEAR compare*” | (indirectly NEAR/2 compare*) (all text) | (indirectly adj2 compare$).tw. |
| 17 | #14 or #15 or #16 | #14 or #15 or #16 | 14 or 15 or 16 |
| 18 | #7 and #13 | #7 and #13 | 7 and 13 |
| 19 | #17 and #18 | #17 and #18 | 17 and 18 |
