# Supplementary material for: Indirect Comparisons: A Review of Reporting and Methodological Quality
Source: PLoS One. 2010 Nov 10;5(11):e11054. doi: 10.1371/journal.pone.0011054 (PMC2978085; doi:10.1371/journal.pone.0011054)
Supplement: Table S2 — Characteristics of included reviews. *Outcome name (primary outcome or main outcome); data type; measure of effect. **NT: number of trials; NP: number of patients. (0.21 MB DOC) [file pone.0011054.s002.doc]

| **Review** | **Patients** | **Outcome*** | **Interventions compared in the indirect comparison** | **Indirect comparisons**** | **Common comparator used in the indirect comparison** |
| --- | --- | --- | --- | --- | --- |
| Abou Setta 2007 [16] | Reproductive: women undergoing embryo transfer. | Live birth rate (main); dichotomous; odds ratio. | Non pharmacological: delivery. | Distance 1 (<7.5 mm) vs. distance 2 (10±2.5mm) (NT=2, NP=1368). | Distance 3.5 (undefined distance). |
| Berner 2006 [17] | Reproductive: men with erectile dysfunction. | Erectile function domain score (main); continuous; mean difference. | Drugs. | 1. Sildenafil vs. Tadalafil (NT=11, NP=2585).  2. Sildenafil vs. Vardenafil (NT=6, NP=3249).  3. Tadalafil vs. Vardenafil (NT=11, NP=3838). | Placebo. |
| Biondi-zoccai 2005 [15] | Circulatory: patients undergoing percutaneous coronary intervention. | Rate of target lesion revascularization at 12 months (primary); dichotomous; odds ratio. | Non pharmacological:  Treatment delivery. | 1. Polymeric sirolimus-eluting stent (PSES) vs. Polymeric paclitaxel-eluting stent (PPES) (NT=10, NP=4363).  2. Everolimus-eluting stents vs. PPES (NT=7, NP=2463).  3. Actinomycin-d-eluting stents vs. PPES (NT=6, NP=2717).  4. Mycophenolate-eluting stent vs. PPES (NT=6, NP=2507).  5. Apolymeric paclitaxel-eluting stent vs. PPES (NT=9, NP=3467).  6. Everolimus-eluting stents vs. PSES (NT=7, NP=2112).  7. Actinomycin-d-eluting stents vs. PSES (NT=6, NP=2366).  8. Mycophenolate-eluting stent vs. PSES (NT=6, NP=2156).  9. Apolymeric paclitaxel-eluting stent vs. PSES (NT=9, NP=6152). | Bare–metal stents. |
| Boonen 2007 [18] | Musculo-skeletal: post-menopausal women and men aged 50 years and over. | Hip fracture (primary); dichotomous; risk ratio. | Non pharmacological:  supplements. | Vitamin D and calcium vs. vitamin D alone (NT=10, NP=54592). | Placebo or no treatment. |
| Brown 2006 [19] | Gastrointestinal: preventing gastrointestinal toxicity caused by NSAIDS used to treat pain in adults. | Serious gastrointestinal complications (primary); dichotomous; risk ratio. | Drug combinations. | 1. histamine-2 receptor antagonist (H2RA) plus non-steroidal anti-inflammatory drug (NSAID) vs. PPI (proton pump inhibitor) plus NSAID, (NT=8, NP=2002).  2. H2RA plus NSAID vs. misoprostol plus NSAID, (NT=14, NP=12401).  3. H2RA plus NSAID vs. Cox-2 coxib NSAID, (NT=15, NP=22348).  4. PPI plus NSAID vs. misoprostol plus NSAID, (NT=14, 12615).  5. PPI plus NSAID vs. Cox-2 coxib NSAID, (NT= 15, NP=22562).  6. Misoprostal plus NSAID vs. Cox-2 coxib NSAID, (NT=21, NP=32961).  7. H2RA plus NSAID vs. Cox-2 preferential NSAID, (NT=23, NP=23619).  8. PPI plus NSAID vs. Cox-2 preferential NSAID,(NT=23, NP=23833).  9. Misoprostal plus NSAID vs. Cox-2 preferential NSAID, (NT=29, NP=34232).  10. Cox-2 coxib NSAID vs. Cox-2 preferential NSAID, (NT=30, NP=44179). | NSAIDs. |
| Buscemi 2007 [20] | Other: chronic insomnia in adults. | Sleep onset latency (main); continuous; mean difference. | Drugs. | 1. Benzodiazepine vs. non-benzodiazepine (NT=91, NP=16563).  2. Antidepressant vs. non-benzodiazepine (NT=52, NP=11854).  3. Antidepressant vs. Benzodiazepine (NT=55, NP=6455). | Placebo. |
| Buttner 2004 [53] | Post-operations: post-operative nausea and vomiting. | Nausea (main); dichotomous; risk ratio. | Drug doses. | Many different doses of haloperidol compared (unclear numbers of trials and patients). | Placebo. |
| Chou 2006 [21] | HIV: HIV infected patients. | Death or disease progression (main); dichotomous; odds ratio. | Drug combinations. | Two nucleoside reverse transcriptase inhibitors (NRTIs) plus one non-nucleoside reverse transcriptase inhibitors (NNRTI) vs. two NRTIs plus one protease inhibitor (NT=14, NP=4042). | Two NRTIs. |
| Clark 2004 [22] | Musculo-skeletal:  adults with rheumatoid arthritis. | ACR 20 (main); dichotomous; risk difference. | Drugs. | 1. Anakinra plus methotrexate vs. [Tumor necrosis factor](http://en.wikipedia.org/wiki/Tumor_necrosis_factor) inhibitor plus methotrexate (NT=unclear, NP=unclear). | Methotrexate. |
| Collins 2007 [23] | Cancer: men with hormone-refractory metastatic prostate cancer. | Death (main); time to event; hazard ratio. | Drug combinations. | Docetaxel plus prednisone versus prednisone (NT= 4, NP=1529). | Mitoxantrone plus prednisone. |
| Coomarasamy 2003 [24] | Reproductive: women with threat of, or actual, preterm labour. | Delivery delay by at least 48 hours (main); dichotomous; odds ratio. | Drugs. | Nifedipine vs. Atosiban (NT= 13, NP=1531). | β-agonists. |
| Costa 2005 [59] | Musculo-skeletal: patients with idiopathic cervical dystonia. | Improvement in symptomatic rating scales (primary); dichotomous; odds ratio. | Drugs. | Dysport vs. Botox (NT= 13, NP=680). | Placebo. |
| Dolovich 2000 [25] | Circulatory: adults with venous thromboembolism. | Recurrent venous thromboembolism (main); dichotomous; risk ratio. | Drug doses. | 1. Once daily low-molecular-weight heparins (LMWH) vs. twice daily LMWH (NT=13, NP=4447).  2. Inpatient LMWH vs. outpatient LMWH, (NT=13, NP=4447). | Unfractionated heparin. |
| Eckert 2006 [26] | Psychological: adult outpatients with major depressive disorder. | Depression rating scale (main); continuous; Hedge's G. | Drugs. | Venlafaxine vs. escalopram (NT=10, NP=2724). | Placebo. |
| Einarson 2000 [27] | Ocular: patients with open angle glaucoma. | Intraocular pressure (main); continuous; mean difference. | Drugs. | Latanoprost vs. Brimonidine. Sub-grouped at 3 months (NT=10, NP=975) and 6 months (NT= 7, NP=739). | Control. |
| Gisbert 2000 [28] | Gastrointestinal: peptic ulcer or non-ulcer dyspepsia. | Eradication of helicobacter pylori (main); dichotomous; odds ratio. | Drug combinations. | 1. Proton pump inhibitor plus two antibiotics (Clarithromycin plus Amoxycillin) vs. proton pump inhibitor plus two antibiotics (Clarithromycin plus Nitroimidazole), (NT= 14, NP=2475).  2. Ranitidine bismuth citrate (RBC) plus two antibiotics (Clarithromycin plus Amoxycillin) vs. RBC plus two antibiotics (Clarithromycin plus Nitroimidazole),(NT=14, NP=2475). | Unadjusted (naive) indirect comparison. |
| Habib 2004 [29] | Post-operations: post-operative nausea and vomiting. | Nausea (primary); dichotomous; risk ratio. | Drug combinations. | 5-HT3 receptor antagonist with Droperidol vs. 5-HT3 receptor antagonist with Dexamethasone (NT=7, NP=670). | 5-HT3 receptor antagonist. |
| Hind 2003 [30] | Circulatory: scheduled for central venous access. | Number of failed catheter placements (main); dichotomous; risk ratio. | Non-pharmacological: methods. | 2D ultrasound guidance vs. doppler ultrasound guidance (NT=16, NP=1646). | Landmark method. |
| Hochberg 2003 [31] | Musculo-skeletal: active rheumatoid arthritis. | American College of Rheumatology 20 response (main); dichotomous; risk ratio. | Drugs. | 1. Etanercept vs. Infliximab (NT= 2, NP=517).  2. Adalimumab vs. Etanercept (NT=3, NP=625).  3. Infliximab vs. Adalimumab (NT= 3, NP=964). | Placebo. |
| Indolfi 2005 [14] | Circulatory: coronary artery disease. | Major adverse clinical events -death, myocardial infarction, coronary artery bypass grafting, target vessel, target lesion revascularization (main); dichotomous; risk ratio. | Non pharmacological:  Treatment delivery. | Sirolimus eluting stents vs. paclitaxel eluting stents (NT= 13, NP=3860). | Bare-metal stents. |
| Jones 2004 [32] | Circulatory: patients with established peripheral arterial disease or those with a history of myocardial infarction, ischaemic stroke or transient ischaemic attacks. | Myocardial infarction (main); dichotomous; risk ratio. | Drugs. | 1. Clopidogrel vs. MR-dipyridamole (NT= 2, NP=22488).  2. Clopidogrel vs.MR-dipyridamole plus aspirin (NT= 2, NP=22484). | Aspirin. |
| Li Wan Po 1997 [33] | Post-operative/ musculo-skeletal: patients with postsurgical pain, arthritis, and musculoskeletal pain. | Pain intensity (main); continuous; mean difference. | Drug combinations. | Paracetamol vs. Paracetamol and Dextropropoxyphene (NT=26, NP=1541). | Placebo. |
| Lim 2003 [34] | Circulatory: prevention of occlusion of vein grafts in patients who have undergone coronary surgery. | Graft occlusion (main); dichotomous; risk ratio. | Drug doses. | Medium Aspirin dose vs. low Aspirin dose (NT= 5, NP=1356). | Placebo. |
| Lowenthal 1994 [35] | Circulatory: patients following cerebrovascular accident-stroke or transient ischaemic attack. | Death (main); dichotomous; risk reduction. | Drug combinations. | Aspirin vs. Aspirin plus Dipyridamole (NT= 9, NP=8397). | Placebo. |
| Mason 2004 [36] | Musculo-skeletal: acute pain resulting from any strains, sprains or sports injuries. | Clinical success-50% or more reduction in pain (main); dichotomous; risk ratio. | Drugs. | Types of non-steroidal anti-inflammatory drugs (Ketoprofen, Ibuprofen, Felbinac, Piroxicam, Indomethacin) compared with each other (number of patients and trials varies with comparisons). | Placebo. |
| McAlister 2004 [37] | Circulatory: patients with heart failure. | All cause mortality (main); dichotomous; risk ratio. | Non-pharmacological: methods of delivery of care. | 1. Multidisciplinary teams providing specialized follow-up in heart failure clinics or non-clinic settings vs. enhanced self-care activities alone, (NT=unclear, NP=unclear).  2. Multidisciplinary teams providing specialized follow-up in heart failure clinics or non-clinic settings vs. programs that employed regular telephone contact and follow-up with primary care practitioners (NT=18, NP=4569). | Usual care. |
| McLeod 2007 [38] | Musculo-skeletal: adults with active ankylosing spondylitis | Assessment in ankylosing spondylitis (main); dichotomous; risk ratio. | Drugs. | 1. Adalimumab vs. Etanercept (NT= 6, NP=998).  2. Adalimumab vs. Infliximab (NT=4, NP=745).  3. Etanercept vs. Infliximab (NT= 6, NP=949). | Placebo. |
| Mudge 2005 [39] | Psychiatric: schizophrenia. | Clinical response (main); dichotomous; odds ratio. | Drugs. | Risperidone vs. Olanzapine (NT=22, NP=5533). | Haloperidol. |
| Norris 2007 [40] | Diabetes: adults with type two diabetes, pre-diabetes, metabolic syndrome. | Blood glucose (primary); continuous; mean difference. | Drugs. | Pioglitazone vs. Rosiglitazone (NT= 37, NP=10423). | Placebo. |
| Otoul 2005 [41] | Other: patients with drug-resistant partial epilepsy (in some trials). | Seizure reduction (50% responder rate) (main); dichotomous; odds ratio. | Drugs. | 1. Levetiracetam vs. Gabapentin (NT=8, NP=1901).  2. Levetiracetam vs. Lamotrigine (NT= 14, NP=2147).  3. Levetiracetam vs. Oxcarbazepine (NT= 5, NP=1865).  4. Levetiracetam vs. Tiagabine (NT= 6, NP=1673).  5. Levetiracetam vs. Topiramate (NP=12, NP=1953).  6. Levetiracetam vs. Zonisamide (NP=6, NP=1403). | Placebo. |
| Otto 2001  [42] | Psychological: panic disorder with or without agoraphobia. | Unclear outcome (main); continuous; standardized mean difference. | Drugs. | 1. Serotonin selective reuptake inhibitors vs. Antidepressant (NT=unclear, NP=unclear).  2. Serotonin selective reuptake inhibitors vs. imipramine (NT=unclear, NP=unclear). | Placebo. |
| Panidou 2004 [43] | HIV: hiv-1- infected patients. | HIV-1 ribonucleic acid level (main); dichotomous; risk difference. | Non-pharmacological: testing methods. | Virtual phenotypic resistance testing vs. control (NT= 6, NP=1460). | Phenotypic resistance testing. |
| Pignon 1992 [44] | Cancer: patients with small cell cancer. | Death (main); dichotomous; risk ratio. | Treatment regimen. | 1. Chemotheraphy plus early radiotherapy vs. Chemotheraphy plus late radiotherapy (NT=unclear, NP=unclear).  2. Chemotheraphy with sequential radiotherapy vs. Chemotheraphy without sequential radiotherapy (NT=unclear, NP=unclear).  3. Men vs. women (NT=13, NP=2101).  4. Performance status of one vs. performance status of 0, (NT=13, NP=2045). | Chemotheraphy. |
| Richy 2005 [45] | Musculo-skeletal: osteoporosis. | Bone mineral density; (main); continuous; cohen's D. | Non pharmacological:  supplements. | Native vitamin D vs. Calcitriol or Alfacalcidol (NT= 32, NP=14523). | Control. |
| Rocha 2000 [46] | Circulatory: patients with deep venous thrombosis. | Clot reduction (main); dichotomous; odds ratio. | Drug doses. | Two doses of low molecular weight heparin (LMWH) vs. one dose of LMWH (NT= 12, NP=1423). | Unfractionated heparin. |
| Sanchez- Ramos 2002 [13] | Reproductive: labour induction. | Tachysystole (main); dichotomous; odds ratio. | Drug doses. | Intravaginal misoprostol 25 mcg vs. intravaginal misoprostol 50 cg (NT= 2, NP=206). | Unadjusted (naive) indirect comparison. |
| Sauriol 2001 [47] | Psychiatric: Schizophrenia. | Brief Psychiatric rating scale total score change (main); continuous; mean difference. | Drugs. | Olanzapine vs. Risperidone (NT= 11, NP=3552). | Haloperidol. |
| Stettler 2006 [48] | Circulatory: patients with angina and signs of myocardial ischaemia. | In-stent restenosis  (main); count; incidence rate ratio. | Non pharmacological: treatment delivery. | Sirolimus eluting stents vs. paclitaxel eluting stent (NT= 10, NP=4513). | Bare metal stents. |
| Vestergaard 2007 [12] | Musculo-skeletal: secondary osteoporosis. | Vertebral fractures  (main); dichotomous;  risk ratio. | Treatment regimens. | 1. Treatment duration <18 months vs. >18 months (NT= 7, NP=4359).  2. Parathyroid hormone (PTH) plus HRT vs. PTH alone (NT= 6, NP=4251).  3. PTH (1-84) vs. PTH (1-34) (NT= 2, NP=3857). | Control. |
| Vis 2005 [49] | Psychological: major depressive disorder. | Remission (primary); dichotomous; risk difference. | Drugs. | Duloxetine vs. Venlafaxine-XR (NT= 9, NP=2114). | Placebo. |
| Wu 2006 [50] | Other: smokers. | Smoking cessation at one year (primary); dichotomous; odds ratio. | Drugs. | 1. Bupropion vs. nicotine replacement therapy (NT= 60, NP=26660).  2. Varenicline vs. nicotine replacement therapy (NT=53, NP=24040). | Placebo or control. |
| Yazdanpanah 2004 [51] | HIV: HIV positive adults. | Progression to aids or death (primary); dichotomous; odds ratio. | Drugs. | Two nucleoside reverse transcriptase inhibitors (NRTIs) and a protease inhibitor vs. two NRTIs and a non-nucleoside analogue reverse transcriptase inhibitor (NNRTI) (NT=14, NP=6785). | Two NRTIs. |
| Zhou 2006 [52] | Circulatory: cardiovascular disease prevention. | Major coronary events (main); dichotomous; risk ratio. | Drugs. | 1. Provastatin vs. Simvastatin (NT= 6, NP=50552).  2. Atorvastatin vs. Simvastatin (NT= 4, NP=38123).  3. Provastatin vs. Atorvastatin (NT= 6, NP=38715). | Placebo. |
