# Supplementary material for: Indirect Comparisons: A Review of Reporting and Methodological Quality
Source: PLoS One. 2010 Nov 10;5(11):e11054. doi: 10.1371/journal.pone.0011054 (PMC2978085; doi:10.1371/journal.pone.0011054)
Supplement: Table S3 — Quality assessment results for individual reviews. Abbreviations: c: comparable/consistent, f: fixed effects model, h: homogeneity reported or determined from results, he: heterogeneity reported or determined from results, n: no, na: not applicable, nc: not comparable/consistent, nr: not reported, nt: no three-arm trials, r: random effects model, t: using a statistical test, u: unclear, y: yes. (0.18 MB DOC) [file pone.0011054.s003.doc]

| CRITERIA  REVIEW | Method | | Similarity | | | | | | Homogeneity | | | Consistency | | | | | | | | Interpretation | | Reporting | | |
| --- | --- | --- | --- | --- | --- | --- | --- | --- | --- | --- | --- | --- | --- | --- | --- | --- | --- | --- | --- | --- | --- | --- | --- | --- |
|  | Adequate indirect comparison method? | If adequate method, treatment effect and measure of precision reported? | State similarity assumption? | Report a method to assess similarity? | Reasonable method to assess similarity? | Characteristics reported? | Characteristics compared? | Characteristics comparable? | Adequate method to assess statistical heterogeneity? | Is the homogeneity assumption satisfied or is heterogeneity accounted for if present? | Adequate method to assess clinical or methodological heterogeneity? | Consistency assessed? | Consistent estimates are combined? | Inconsistency accounted for by not combining? | Characteristics compared? | Characteristics comparable? | Three-arm trials correctly analysed? | Justification given? | Present direct evidence trials results’? | Distinction made between evidence types? | State need direct evidence trials? | Present both meta-analysis results? | Highlight indirect comparison? | Trials’ results reported? |
| Abou-Setta 2007 | y | y | n | n | u | y | n | nr | na | na | na | na | na | na | na | na | na | na | na | y | y | y | y | n |
| Berner 2006 | y | n | y | n | y | y | n | nr | y | h/f | h | na | na | na | na | na | na | na | na | n | y | y | n | y |
| Biondi-zoccai 2005 | y | y | n | n | y | y | n | nr | y | he/r | n | y | y | c | n | nr | nt | y | n | y | y | n | n | n |
| Boonen 2007 | y | y | y | n | n | y | n | nr | y | h/r | h | y | nc | y | y | n | n | y | n | y | n | y | y | y |
| Brown 2006 | y | y | n | n | n | y | n | nr | y | he/r | y | y | n | c | n | nr | y | y | y | y | y | y | y | y |
| Buscemi 2007 | y | y | n | n | y | y | n | nr | y | he/u | y | n | u | u | n | nr | n | n | n | y | y | y | y | y |
| Buttner 2004 | y | n | n | n | y | y | n | nr | u | he/f | y | n | u | u | n | nr | n | n | n | n | n | y | n | y |
| Chou 2006 | y | y | y | n | y | y | n | nr | y | he/r | n | y, t | nc | y | y | u | y | y | y | y | n | y | y | y |
| Clark 2004 | y | y | y | n | n | n | y | y | n | u/u | n | na | na | na | na | na | na | na | na | y | y | y | n | n |
| Collins 2007 | y | y | y | n | y | y | y | n | y | h/f,r | h | na | na | na | na | na | na | na | na | y | n | y | y | y |
| Coomarasamy 2003 | y | y | n | n | n | n | n | nr | y | h/r | h | na | na | na | na | na | na | na | na | y | y | y | y | n |
| Costa 2005 | y | n | n | n | y | y | n | nr | n | u/f | n | na | na | na | na | na | na | na | na | n | y | n | n | y |
| Dolovich 2000 | y | n | n | n | n | y | n | nr | y | h/r | h | na | na | na | na | na | na | na | na | n | y | y | n | n |
| Eckert 2006 | y | y | n | n | y | y | y | nr | n | u/u | n | n | u | u | y | y | n | y | y | y | n | n | y | y |
| Einarson 2000 | y | n | n | n | n | y | n | nr | u | u/r | n | na | na | na | na | na | na | na | na | y | y | y | n | y |
| Gisbert 2000 | n | na | n | n | n | y | n | nr | y | he/f | n | n | u | u | n | nr | n | n | n | n | n | y | n | y |
| Habib 2004 | y | n | n | n | y | y | n | nr | y | he/r | y | na | na | na | na | na | na | na | na | n | n | y | n | y |
| Hind 2003 | y | y | n | n | y | y | n | nr | n | u/u | y | na | na | na | na | na | na | na | na | n | n | y | n | n |
| Hochberg 2003 | y | y | y | n | n | y | y | y | n | u/f | n | na | na | na | na | na | na | na | na | y | n | n | y | n |
| Indolfi 2005 | y | n | n | n | y | y | n | nr | y | h/r | h | na | na | na | na | na | na | na | na | y | y | y | n | y |
| Jones 2004 | y | y | y | n | n | y | y | n | na | na | na | na | na | na | na | na | na | na | na | y | y | y | y | y |
| Li Wan Po 1997 | y | n | n | n | n | y | n | nr | y | he/r | y | n | u | u | n | nr | n | n | y | n | n | y | y | n |
| Lim 2003 | y | y | y | n | n | y | y | n | n | u/u | n | na | na | na | na | na | na | na | na | y | n | y | n | y |
| Lowenthal 1994 | y | n | n | n | n | y | n | nr | y | h/f | h | n | u | u | n | nr | n | n | y | n | y | y | y | n |
| Mason 2004 | y | n | n | n | n | y | n | nr | y | he/f | n | y | n | c | n | nr | y | n | y | n | n | y | y | y |
| McAlister 2004 | y | y | n | n | u | y | n | nr | n | u/r | u | na | na | na | na | na | na | na | na | n | y | y | y | y |
| McLeod 2007 | y | y | n | n | y | y | n | nr | y | he/f,r | y | na | na | na | na | na | na | na | na | y | n | y | y | y |
| Mudge 2005 | y | n | n | n | y | y | y | u | y | u/r | n | n | u | u | y | u | n | y | y | y | n | n | y | n |
| Norris 2007 | y | y | n | n | y | y | n | nr | y | he/r | y | n | u | u | n | nr | nt | y | n | y | y | y | y | n |
| Otoul 2005 | y | y | y | n | n | n | n | nr | y | h/f | h | na | na | na | na | na | na | na | na | n | y | y | y | n |
| Otto 2001 | y | n | n | n | n | n | n | nr | n | u/u | n | na | na | na | na | na | na | na | na | n | y | y | n | n |
| Panidou 2004 | y | y | n | n | y | y | n | nr | y | he/r | y | na | na | na | na | na | na | na | na | n | n | y | y | n |
| Pignon 1992 | y | n | n | n | n | y | n | nr | n | u/f | n | na | na | na | na | na | na | na | na | y | y | y | n | n |
| Richy 2005 | y | y | n | n | n | y | n | nr | u | u/f,r | y | n | u | u | n | nr | nt | n | y | y | y | y | n | n |
| Rocha 2000 | y | n | n | n | n | y | n | nr | n | u/u | n | na | na | na | na | na | na | na | na | n | n | y | n | n |
| Sanchez- Ramos 2002 | n | na | n | n | n | n | y | y | na | na | na | y | n | c | n | nr | nt | n | y | n | n | n | n | n |
| Sauriol 2001 | y | y | y | n | n | y | y | n | u | u/f | n | n | u | u | y | y | nt | y | y | y | y | y | y | n |
| Stettler 2006 | y | y | n | n | y | y | y | y | y | he/r | n | na | na | na | na | na | na | na | na | y | n | y | y | y |
| Vestergaard 2007 | y | n | n | n | n | y | n | nr | n | u/u | n | na | na | na | na | na | na | na | na | n | y | y | n | y |
| Vis 2005 | y | n | n | n | y | y | n | nr | n | u/r | y | na | na | na | na | na | na | na | na | n | y | y | n | n |
| Wu 2006 | y | y | n | n | n | y | n | nr | y | he/r | n | n | u | u | n | nr | n | n | y | y | n | y | y | y |
| Yazdanpanah 2004 | y | y | n | n | y | y | y | n | y | u/r | n | na | na | na | na | na | na | na | na | y | y | y | y | y |
| Zhou 2006 | y | y | y | n | y | y | n | u | y | he/f,r | y | na | na | na | na | na | na | na | na | y | y | y | y | y |
